# Swimming kinematics and performance of spinal transected lampreys with different levels of axon regeneration

**DOI:** 10.1242/jeb.242639

**Published:** 2021-11-05

**Authors:** Jacob Fies, Brad J. Gemmell, Stephanie M. Fogerson, Jennifer R. Morgan, Eric D. Tytell, Sean P. Colin

**Affiliations:** 1 Marine Biology and Environmental Science, Roger Williams University, Bristol, RI 02809 USA; 2 Integrative Biology, University of South Florida, Tampa, FL 33620 USA; 3 The Eugene Bell Center for Regenerative Biology and Tissue Engineering, Marine Biological Laboratory, Woods Hole, MA 02543 USA; 4 Department of Biology, Duke University, Durham, NC 27708 USA; 5 Department of Biology, Tufts University, Medford, MA 02155 USA

**Keywords:** Anguilliform, Neuromuscular, *Petromyzon marinus*

## Abstract

Axon regeneration is critical for restoring neural function after spinal cord injury. This has prompted a series of studies on the neural and functional recovery of lampreys after spinal cord transection. Despite this, there are still many basic questions remaining about how much functional recovery depends on axon regeneration. Our goal was to examine how swimming performance is related to degree of axon regeneration in lampreys recovering from spinal cord transection by quantifying the relationship between swimming performance and percent axon regeneration of transected lampreys after 11 weeks of recovery. We found that while swimming speeds varied, they did not relate to percent axon regeneration. In fact, swimming speeds were highly variable within individuals, meaning that most individuals could swim at both moderate and slow speeds, regardless of percent axon regeneration. However, none of the transected individuals were able to swim as fast as the control lampreys. To swim fast, control lampreys generated high amplitude body waves with long wavelengths. Transected lampreys generated body waves with lower amplitude and shorter wavelengths than controls, and to compensate, transected lampreys increased their wave frequencies to swim faster. As a result, transected lampreys had significantly higher frequencies than control lampreys at comparable swimming velocities. These data suggest that the control lampreys swam more efficiently than transected lampreys. In conclusion, there appears to be a minimal recovery threshold in terms of percent axon regeneration required for lampreys to be capable of swimming; however, there also seems to be a limit to how much they can behaviorally recover.

## Abstract (precis)

**Summary:** Lampreys that have recovered from having their spinal cords transected do not fully regain swimming abilities and are not able to swim as efficiently as non-transected lampreys.

## INTRODUCTION

Spinal cord injury in mammals, including humans, leads to permanent loss of movement and sensation because the regeneration of damaged or lost axons within the central nervous system is limited. In contrast, in many non-mammalian vertebrates such as lampreys, fishes and amphibians, spinal axons undergo robust spontaneous regeneration, leading to functional recovery even after a complete spinal cord transection (Haspel et al., 2021; Morgan and Shifman, 2014; Rasmussen and Sagasti, 2017). Amongst the highly regenerative species, lampreys (family Petromyzontidae) have become a leading model for the study of neural mechanisms of spinal cord regeneration over the last 50 years owing to the robustness and reproducibility with which behavioral recovery occurs after injury, combined with the ability to image long-distance regeneration of descending reticulospinal (RS) axons and to perform electrophysiology recordings from regenerating neurons (Rovainen, 1976; Selzer, 1978; Cohen et al., 1986; Cooke and Parker, 2009; Oliphint et al., 2010; Parker, 2017; Hanslik et al., 2019). These features of the lamprey spinal cord injury model permit a detailed examination of the neural mechanisms underlying behavioral recovery of locomotion to a degree that surprisingly has not yet been reported in other commonly used spinal cord regeneration models, including zebrafish (Haspel et al., 2021). In particular, our understanding of the recovery of locomotor behaviors, including swimming and burrowing, is at present much farther advanced in the lamprey model (Cohen et al., 1986; McClellan, 1990, 1994; Davis and McClellan, 1994; Oliphint et al., 2010; Hanslik et al., 2019; Katz et al., 2020). Yet, we still do not fully understand how behavioral recovery relates to regeneration of descending RS axons or plasticity within other neuronal subtypes comprising the spinal locomotor circuits.

Larval sea lampreys (*Petromyzon marinus*) are well-characterized anguilliform swimmers (McClellan et al., 2016). Based on measurements from eels, another anguilliform swimmer, it has been suggested that this swimming mode is highly efficient (Van Ginneken et al., 2005). This form of propulsion is characterized by a traveling wave that moves from the head to the tail with a relatively short wavelength, so that about a full wave is present on the body at any time. The amplitude of this traveling wave increases as it travels down the body (Lauder and Tytell, 2005). These kinematics interact with the adjacent fluid to slowly build fluid vorticity and strong negative pressure regions that serve to efficiently generate a suction thrust that pulls the anguilliform swimmer forward (Gemmell et al., 2016).

Healthy lampreys generate the characteristic traveling wave using muscle contractions along the side of their body that are initiated just caudal to the head and travel toward the tail. By alternating these contractions on each side of the body, the lamprey can generate successive traveling waves that make up each swimming cycle (McClellan, 1989; Williams et al., 1989; Williams and McMillen, 2015). The speed of the observed body wave is slower than that of the muscle contraction as a result of the interaction of forces acting on the body, which include the forces generated by the muscles and the resistive forces of the fluid acting on the body (Ding et al., 2012; Tytell et al., 2010; Williams et al., 1989; Williams and McMillen, 2015). Demonstrating the robustness of this behavior, within several months after a complete spinal cord transection, lampreys are able to achieve robust recovery of swimming and burrowing behaviors (Cohen et al., 1999; Hanslik et al., 2019; Katz et al., 2020; McClellan, 1989, 1994; Oliphint et al., 2010; Rovainen, 1976; Selzer, 1978). Remarkably, they can also recover swimming after a second spinal re-transection (Hanslik et al., 2019). Therefore, lampreys have served as a model for studying recovery from spinal cord injuries. Lampreys spontaneously recover swimming behaviors within 8–12 weeks after their spinal cord is transected rostrally at the level of the 5th gill owing, in part, to long-distance regeneration of descending RS axons (McClellan, 1994; Oliphint et al., 2010; Rovainen, 1976). Initially, such transected animals are completely paralyzed (Hanslik et al., 2019; Oliphint et al., 2010). Axon regeneration begins 2 to 3 weeks after spinal cord transection with RS axons beginning to regenerate and some observed locomotor function just caudal to where the spinal cord was transected. Progressively over time, locomotor function can be observed more caudally, and by 8–12 weeks, locomotor activity, neural activity and movement patterns can be similar to those of normal, healthy larval lampreys (Cohen et al., 1986; McClellan, 1994; Oliphint et al., 2010). Despite the ability to regenerate axons, the swimming kinematics and performance of recovered lampreys still differs from non-transected lampreys, with generally slower swim speeds (Oliphint et al., 2010). In addition, only a subset of descending RS axons regenerate, making a few, sparse synaptic connections, implicating compensatory mechanisms in locomotor recovery (Davis and McClellan, 1994; Oliphint et al., 2010; Yin and Selzer, 1983). These compensatory mechanisms include synaptic plasticity between several classes of intraspinal interneurons both above and below the lesion, as well as sensory inputs (Cooke and Parker, 2009; Hoffman and Parker, 2011; Parker, 2017; Hoffman and Parker, 2011). At present, surprisingly little is known about how these individual components integrate at the level of spinal circuits to restore locomotor behaviors (Haspel et al., 2021).

Despite lampreys being well documented as robust regenerators after a rostral spinal cord transection, the extent of descending axon regeneration supporting this behavioral recovery is variable from animal to animal, typically ranging from 30 to 70% (Cohen et al., 1986; Hanslik et al., 2019; Oliphint et al., 2010; Rovainen, 1976; Selzer, 1978). How the extent of RS axon regeneration within an individual relates to its behavioral recovery is unknown. Therefore, to understand the relationship between neural regeneration and behavioral recovery, we quantified the kinematics and swimming abilities of larval lampreys that had different levels of RS axon regeneration at several months post-injury and compared their performance with that of uninjured control lampreys. We hypothesized that a greater degree of RS axon regeneration would result in better swimming performance owing to stronger activation of the spinal locomotor circuits below the lesion by the descending commands.

## MATERIALS AND METHODS

### Spinal cord transections

The primary goal of the study was to evaluate how axon regeneration within spinal-transected lampreys was related to functional recovery of swimming. Maintenance and handling of all lampreys strictly adhered to the guidelines approved by the Institutional Animal Care and Use Committee (IACUC) of the Marine Biological Laboratory, Woods Hole, MA, USA. We performed rostral transections of the spinal cord (at the level of the 5th gill) on *n*=11 lampreys and allowed them to recover for 11 weeks post-injury. Under these conditions, RS axon regeneration ranges from 30 to 70% (Davis and McClellan, 1994; Hanslik et al., 2019). As a control, we performed sham treatments on *n*=3 control lampreys (same surgical procedure but their spinal cord was not cut). We also transected the spinal cords of *n*=3 individuals at the mid-body as a subset as another control for the effect of the location of the lesion.

**Fig. 1.**
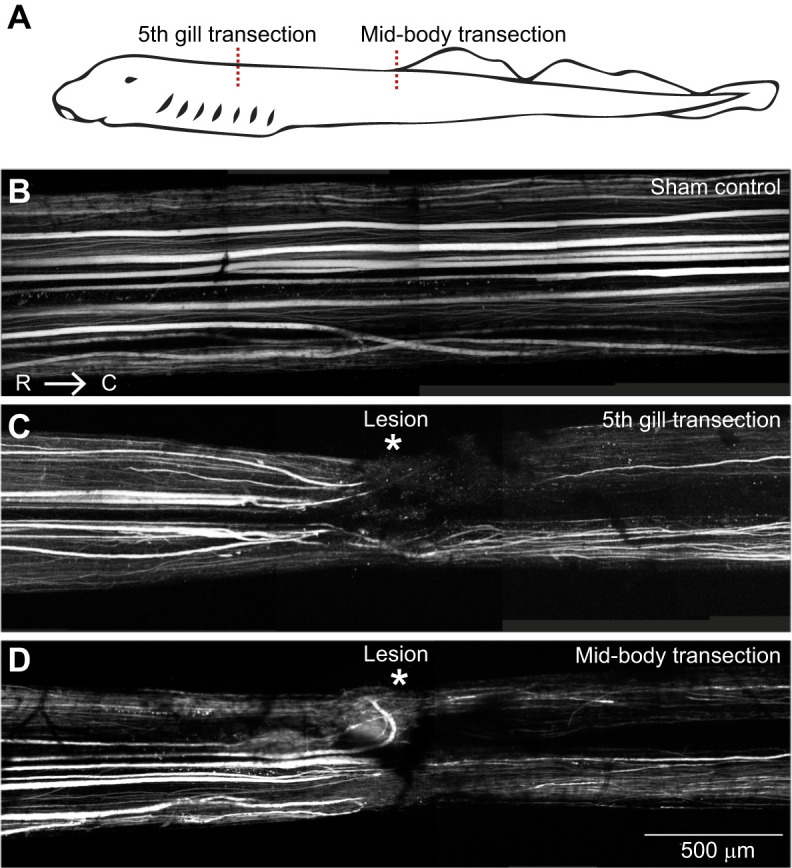
**Reticulospinal (RS) axon regeneration in the lamprey spinal cord.** (A) Schematic of a larval lamprey with the site of spinal cord transection indicated with a red dashed line for a 5th gill or mid-body transection. (B) A montage of confocal z-projections stitched together of a control, sham uninjured spinal cord with axons labeled by a 10 kDa Alexa Fluor 488 dextran, showing fairly uniform labeling along the length of the spinal cord. (C,D) Labeling of axons ∼10.5 weeks post injury in a 5th gill transected and a mid-body transected animal shows sparser axon labeling in the region caudal to the lesion site in comparison to the rostral region, indicating the amount of axon regeneration. Note that the amount of axon regeneration is comparable between the 5th gill and mid-body transected spinal cords. Scale bar in D applies to B–D. Rostral (R) is to the left and caudal (C) is to the right.

All animals used in the experiments were late larval-stage lampreys (*Petromyzon marinus* Linnaeus 1758) [10–14 cm; male and female, though they are sexually undifferentiated at this stage in their life cycle (Ajmani et al., 2021)] that were housed at room temperature (25°C) in 38 l aquariums. Fourteen lampreys (treatments) underwent spinal cord transection surgery (*n*=11 were transected at the 5th gill and *n*=3 at the mid-body) as previously described by Oliphint et al. (2010). Briefly, each lamprey was anesthetized with Finquel MS-222 (0.1 g l^−1^ tank water; Argent Chemical Laboratories) and then placed in a Sylgard-lined Petri dish on a paper towel moistened in oxygenated lamprey Ringer’s solution (in mmol l^−1^: 100 NaCl, 2.1 KCl, 2.6 CaCl_2_, 1.8 MgCl_2_, 4 glucose, 0.5 glutamine, 2 HEPES, pH 7.4). A dorsal incision was made either at the 5th gill or approximately halfway down the length of the body, just above the dorsal fin, through the skin, musculature and fat tissue in order to expose the spinal cord. Then, the spinal cord was completely transected either at the 5th gill or the mid-body with a single horizontal cut made with fine iridectomy scissors (Fig. 1A). The incision was closed with a single suture (Ethilon 6-0 black monofilament nylon, Johnson & Johnson, Langhorn, PA, USA). Three control animals received a sham treatment in which they underwent the same surgical procedures, but the spinal cord was not cut. All animals were then returned to their home tanks for 11 weeks post-injury until they were video-recorded. All procedures were approved by the IACUC at the Marine Biological Laboratory in accordance with the standards set by the National Institutes of Health.

### Video recording and kinematics calculations

At 11 weeks post-injury, videos were taken of the lampreys as they were prompted to swim through a 1.5×5 m acrylic aquarium that was filled with 5 cm of lamprey tank water. Video was captured at 1000 frames s^−1^ using a Photron Fastcam 1024 PCI video camera positioned below the lampreys (as in Gemmell et al., 2016).

To compare the kinematics and swimming of the lamprey, each animal was video-recorded during steady-state swimming following procedures similar to Gemmell et al. (2016) and Du Clos et al. (2019). Accordingly, lampreys were placed at one end of long (1.5 m) tanks, where swimming was initiated by touching the individual gently at the tail. Swimming and kinematics were video-recorded as the lamprey passed the middle of the tank. The rostral position was measured over time throughout the analyzed swim cycles to ensure that the average velocity of the lampresy did not change, indicating that they were no longer accelerating and swimming in steady state. Their bodies were illuminated with a light sheet that was oriented horizontally and directed perpendicular to the camera angle, and the light was generated using two lasers (532 nm, 600 mW continuous wave per laser) placed on opposite sides of the aquarium. Using two lasers eliminated shaded regions around the swimming lampreys and enabled us to thoroughly illuminate the outline of the lampreys. The laser light did not appear to affect the lampreys’ swimming behaviors. Only video sequences where the instantaneous velocities did not deviate from the velocity were averaged over the entire sequence.

Swimming kinematics were quantified manually using ImageJ (NIH) software and an in-house MATLAB program (https://github.com/tytell/neuromech). Raw images of the freely swimming animals were input to a custom program in MATLAB that identified and tracked the midline of the lampreys as they swam. Based on the *X* and *Y* coordinates of the lamprey midline, the maximum amplitude (peak to peak), wavelength and frequency were calculated over time. Maximum amplitude was calculated at the largest amplitude measured in the wave along the body. Wavelength was measured as the distance between wave peaks or troughs.

To arrive at estimates of relative efficiency based on kinematics we calculated Strouhal number (*St*) as *St*=2*fA*/*U*, where *A* is the maximum amplitude, *f* is the frequency and *U* is the swimming speed (Triantafyllou et al., 1993; Tytell, 2004). We also calculated the stride length, the distance traveled per body wave, by dividing swimming speed by wave frequency to get an estimate of how effective each body wave was at propelling the lamprey forward.

### Axon labeling, imaging and regeneration analysis

Following video recording at 10.5 weeks post injury, the descending RS axons were bulk labeled in order to assess the extent of axon regeneration, as previously described (Armstrong et al., 2003; Hanslik et al., 2019; Lau et al., 2013). Briefly, animals were re-anesthetized in MS-222, and a second spinal lesion was made 0.5 cm rostral to the original transection site. A 1×1×1 mm cube of Gelfoam (Pfizer, New York, NY, USA) soaked in 5 mmol l^−1^ Alexa Fluor^®^ 488-conjugated dextran (10 kDa; Thermo Fisher Scientific, Waltham, MA, USA), diluted in lamprey internal solution (180 mmol l^−1^ KCl, 10 mmol l^−1^ HEPES, pH 7.4), was placed in the lesion, which was then closed with a single suture. Spinal cords were harvested 3 days after labeling to allow for maximum transport of dye. The anterograde-labeled, regenerating RS axons were imaged live within whole-mounted spinal cords submerged in oxygenated lamprey Ringer’s solution. Imaging was performed using a Zeiss LSM 510 laser scanning confocal on an Axioskop 2FS upright microscope (10×, 0.3 NA Zeiss EC Plan-Neofluar objective). Z-stacks of spinal cords were acquired at distances ranging from 2 mm proximal to the original transection site to 5 mm distal. Maximum intensity projections were made using the Zeiss LSM software and stitched together in Photoshop (Adobe Photoshop v21.0.0). For 5th gill and mid-body transections, the number of labeled axons crossing fiducial markers positioned at 1.0–1.5 mm proximal and 1.0 mm distal to the transection site were counted. Percent axon regeneration was calculated as the number of labeled axons distal to the transection site, divided by the number of labeled axons at rostral, though we acknowledge that this is a semi-quantitative measurement that may include some smaller regenerating axon branches. Control spinal cords were imaged and analyzed the same way, except without an intervening lesion site.

### Statistics

All comparisons were tested to determine whether they complied with the assumptions of parametric tests. Wave kinematics were compared among treatments (5th gill transected, mid-body transected and sham) using one-way ANOVAs. The relationships between axon regeneration and swimming performance and kinematics were examined using regression analyses. We additionally examined swimming speed as a function of axon regeneration and tail-beat frequency using a mixed model multiple regression, including regeneration, tail-beat frequency and their interaction as fixed factors and individual animal as a random effect. This statistical model was implemented using R 4.0.2 and nlme 3.1-148 (https://CRAN.R-project.org/package=nlme).

## RESULTS

### Axon regeneration after spinal cord injury in lampreys

For this study, we compared axon regeneration, kinematics and performance in lampreys that underwent a rostral spinal cord transection at the level of the 5th gill or alternatively at the mid-body (Fig. 1A). In untransected control spinal cords, RS axons generally projected in relatively straight patterns within the ventromedial and ventrolateral tracts (Fig. 1B). Following a complete spinal cord transection, which severs all the RS axons, the distal portions of the axons degenerate while the proximal axons first retract and then mount a regeneration response (Jin et al., 2009). At ∼10.5 weeks after a rostral spinal cord transection, RS axons proximal to the spinal lesion projected in both straight and curved pathways within the spinal cord (Oliphint et al., 2010), and only a subset of RS axons regenerated distal to the lesion (Fig. 1C). Similar amounts of RS axon regeneration were observed in spinal cords that were transected at the mid-body (Fig. 1D), a perturbation that did not result in paralysis of the animal owing to preservation of the rostral spinal circuits that initiate swimming (Gemmell et al., 2016). To estimate the extent of axon regeneration, we counted the number of Alexa-Fluor^®^ 488-labeled RS axons 1 mm distal to the lesion center and divided this by the number of labeled axons 1–1.5 mm proximal to the lesion. In this cohort of animals, the percent axon regeneration in the spinal cords of individuals was between 33.3 and 84.2% with a median of 58.6%, which is similar to that reported in previous studies (Lau et al., 2013; Oliphint et al., 2010; Yin and Selzer, 1983), thus providing a range of neural regeneration to compare with behavioral performance.

### Swimming performance and kinematics

**Fig. 2.**
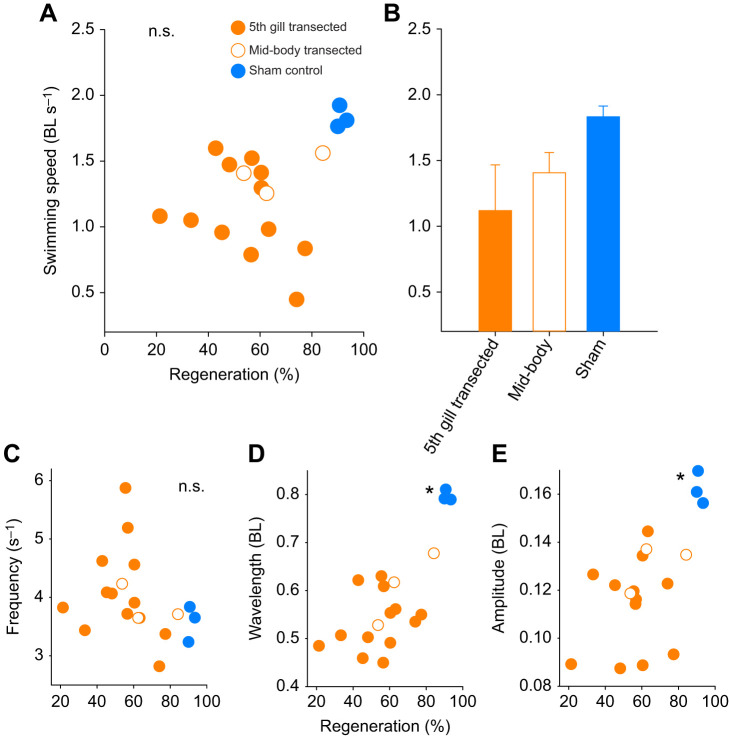
**Comparison of swimming performance and body kinematic parameters of lampreys.** (A) Swimming speed of lampreys versus the degree of axon regeneration (%) after recovering for 10.5 weeks from complete spinal transection (regression analysis, d.f.=1, *P*>0.05). (B) Comparison of mean swimming speeds among treatments (ANOVA, d.f.=2, *P*>0.05). (C–E) Comparison of (C) wave frequency, (D) wavelength and (E) wave amplitude versus the degree of spinal cord regeneration (%; regression including 5th gill and mid-body, d.f.=1, *P*>0.05). Asterisks indicate that the control group kinematics were significantly different than the transected groups (Holm–Šidák *post hoc* comparison, *P*<0.05).

All of the lampreys examined recovered sufficiently to be able to swim. In fact, we found that how fast transected lampreys were capable of swimming was not correlated with the percent of axon regeneration within their spinal cords (regression analysis, d.f.=1, *F*=2.02, *P*=0.2; Fig. 2A). Comparison of swimming speeds among treatments (5th gill transected, mid-body transected and sham control) suggests that the sham control lampreys swam faster [1.83±0.08 body lengths (BL) s^−1^] than transected individuals (1.12±0.35 BL s^−1^), but the differences were not significant (ANOVA, d.f.=2, *F*=2.72, *P*=0.09; Fig. 2B). None of the wave kinematics, including frequency, wavelength and amplitude, were significantly related to axon regeneration for the recovered transected lampreys (regression analysis, d.f.=1, *P*>0.07; Fig. 2C–E).

Comparing control and lesioned animals, regardless of percent regeneration, showed that control sham lampreys had significantly longer wavelengths (5th gill=0.53±0.06 BL; mid-body=0.61±0.08 BL; sham=0.79±0.01 BL) and higher amplitudes (5th gill=0.11±0.02 BL; mid-body=0.13±0.01 BL; sham=0.16±0.01 BL) than those of transected and mid-body lampreys (Holm–Šidák *post hoc* comparison, *P*<0.05), but their wave frequencies (5th gill=4.1±0.81 BL; mid-body=3.7±0.32 BL; sham=3.6±0.31 BL) were not different than those of transected or mid-body lampreys (ANOVA, d.f.=2, *P*>0.1).

**Fig. 3.**
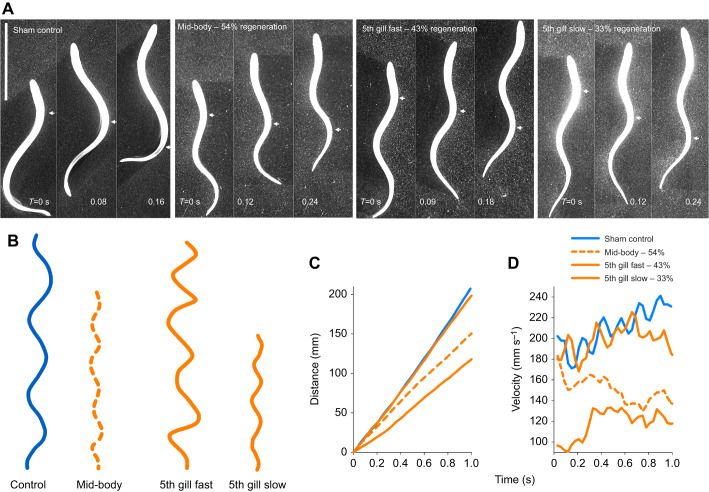
**Comparison of body and swimming kinematics of lampreys.** (A) Sequential images of different lamprey showing the progression of a body wave (indicated by white arrow) moving from head to tail. Notice the control lamprey has only one large wave traveling along the body at a time, whereas all the transected lampreys, regardless of swimming velocity (see D), have multiple smaller waves moving along the body. (B) Tracking of the movement of the head of the lamprey for 1 s. Notice the distance traveled and the evenness versus unevenness of the lateral motion of the heads through time. (C) Distance the different lampreys traveled over 1 s. (D) Velocity of the different lampreys over 1 s. Notice the regular swim cycles of the control lampreys (blue) versus the more erratic motion of the transected lampreys (orange).

Visual comparison of the swimming videos illustrates the differences in wavelength and amplitude between control and lesioned animals. Sequential images of the lampreys (Fig. 3A) reveal that the wavelength of the body wave of the control lamprey was large compared with that of the recovered, transected lampreys. As such, more waves occurred along the bodies of the transected lampreys (1.9±0.2 waves per body) at any one time than the control lampreys (1.2±0.01). The swimming kinematics of the control lampreys were also very regular during consecutive swimming cycles, while the swimming kinematics of the transected lampreys were much more irregular (seen in the motion of the head and swimming velocity; Fig. 3B,D). A fast swimming 5th gill transected individual was included in the comparison to illustrate the differences in the kinematics between the fast transected and the control lampreys (Fig. 3). Despite traveling a similar distance as the controls (Fig. 3C), the transected lampreys still had a smaller wavelength (Fig. 3A), the head moved back and forth much more frequently (Fig. 3B) and the swim pattern was much more erratic than the controls (Fig. 3D).

**Fig. 4.**
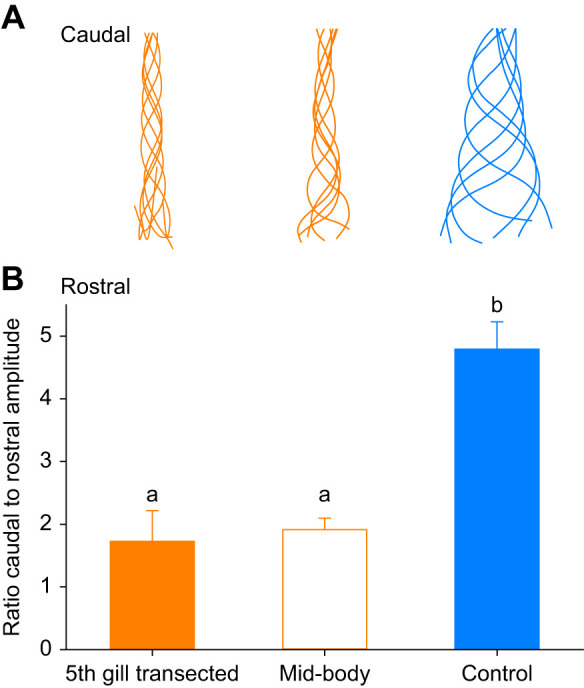
**Change in body wave amplitude as the wave travels from head to tail.** (A) Change in midline of representative lampreys over time. (B) Mean change in amplitude [as ratio of amplitude at the tail (caudal) and the head (rostral)] among treatments. Lowercase letters indicate significantly different treatment groups (Holm–Šidák *post hoc* comparison, *P*<0.05).

A closer look at the wave amplitudes among the groups revealed that the larger amplitudes observed for the control lampreys were achieved by the lampreys increasing amplitude as the wave traveled from head to tail (Fig. 4A). In contrast, the wave amplitudes of the 5th gill and mid-body transected lampreys did not change as much as the waves moved from head to tail. The ratio of the wave amplitude at the head over the amplitude at the tail shows that the amplitude of the control lampreys (4.8±0.43) increased significantly more than the amplitude of the 5th gill (1.7±0.49) and mid-body (1.9±0.18) transected lampreys (Holm–Šidák *post hoc* comparison, *P*<0.05; Fig. 4B).

**Fig. 5.**
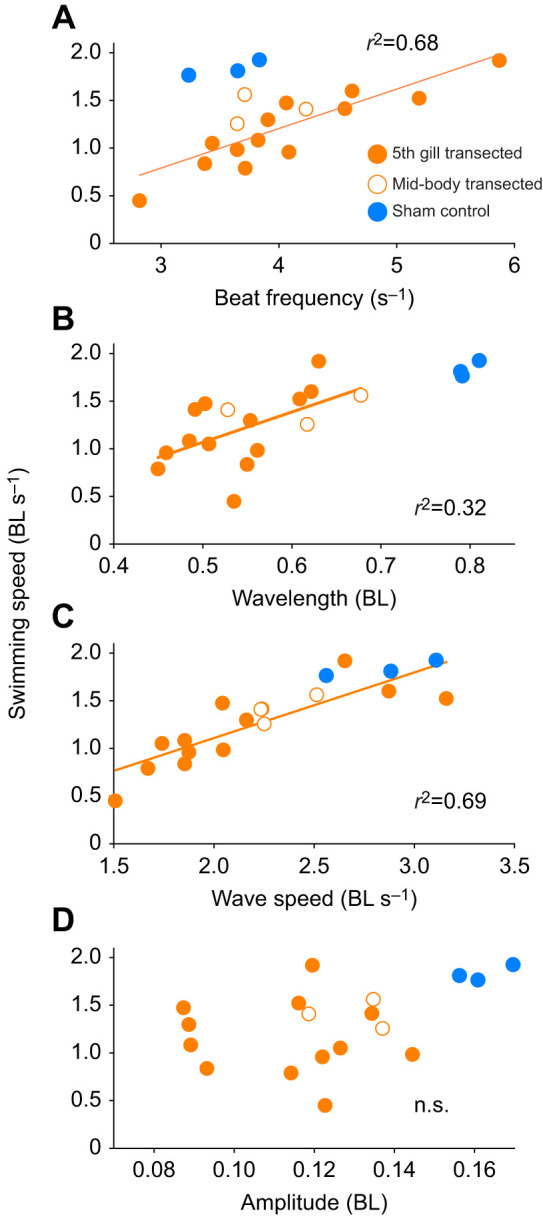
**Body kinematic variables versus swimming speeds of 5th gill transected (filled orange circles), mid-body transected (open circles) and control lampreys (filled blue circles).** (A) Tail-beat frequency, (B) wavelength and (C) wave speed were all positively related to swimming speed for the transected lampreys (regression analysis, *P*<0.01). (D) Wave amplitude of the traveling body waves was not significantly related to swimming speed (regression analysis, *P*>0.1).

Although average swimming speed was not significantly related to axon regeneration, swimming speed was directly related to the body wave characteristics of tail beat frequency, wavelength and wave speed (regression analysis, d.f.=1, *P*<0.01; Fig. 5). Multiple regression indicates that swimming speed depends on tail-beat frequency (*P*<0.001), but not on regeneration percentage (*P*=0.65) or its interaction with tail-beat frequency (*P*=0.88) (Table S1, Fig. S1). Wave amplitude did not have a significant effect on swimming speed (regression analysis, d.f.=1, *P*>0.05; Fig. 5D). The tail-beat frequency of the control sham lampreys was low compared with that of transected lampreys swimming at a similar speed; therefore, the control lampreys were able to achieve higher swimming speeds at lower tail-beat frequencies and wave speeds than the transected lampreys (Fig. 5A,C) (Oliphint et al., 2010).

### Kinematic indicators of swimming efficiency

**Fig. 6.**
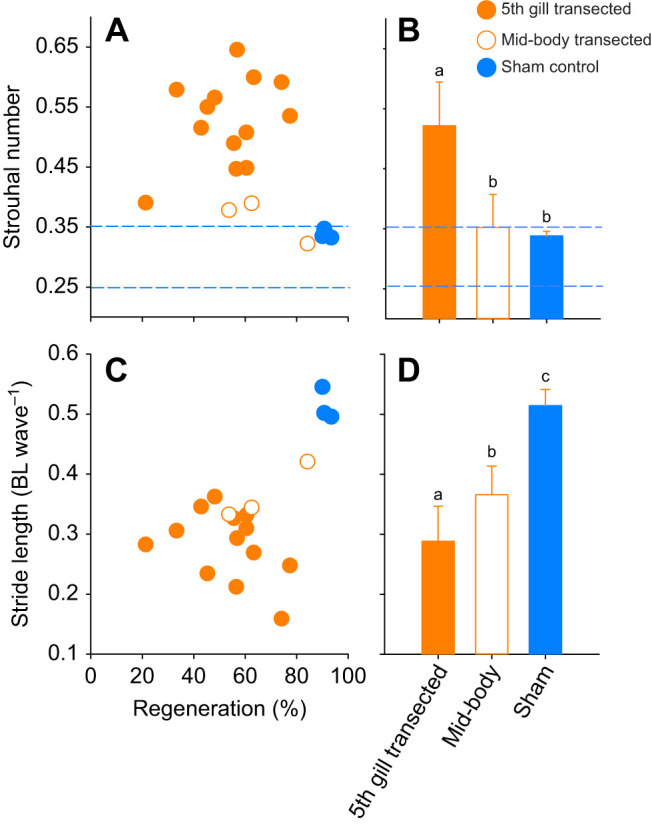
**Effects of percent regeneration on Strouhal number (*St*) and stride length (body lengths traveled per wave) for transected and control lampreys.** (A) *St* of lampreys with different levels of regenerated spinal cord. Dotted blue lines highlight the region where studies have shown animals and flapping foils to have the highest propulsive efficiency. The 5th gill lampreys fell outside the optimal range of *St*, whereas the control and the one mid-body lamprey fall within the optimal range. (B) The 5th gill transected lampreys had significantly higher *St* than the mid-body transected and control lampreys (Holm–Šidák *post hoc* comparison, *P*<0.05). (C) Stride length of lampreys with different levels of regenerated spinal cord. (D) Comparison of the stride lengths among treatments; different lowercase letters designate significantly different groups (Holm–Šidák *post hoc* comparison, *P*<0.05).

To examine how the differences in kinematics and performance may translate into efficiency, we calculated Strouhal number and stride length, indices that can be used as indicators of efficiency (Fig. 6). The *St* of the control lampreys (and one lamprey with a mid-body lesion) fell within the range (*St*=0.25–0.35) that has been shown to provide the maximum propulsive efficiency (Fig. 6A) (Taylor et al., 2003; Eloy, 2012) and were significantly lower than the *St* of the 5th gill transected lampreys (Holm–Šidák *post hoc* comparison, *P*<0.05; Fig. 6B). However, the controls did not significantly differ from the mid-body transected lampreys (Holm–Šidák *post hoc* comparison, *P*>0.05). The control lampreys also swam further with each tail beat (one-way ANOVA, *F*=39.8, *P*<0.001; Fig. 6C). Therefore, both of these indices suggest that even when transected lampreys swim as fast as controls, they do not swim as efficiently.

### Comparison between 5th gill and mid-body transected lampreys

Lampreys that have spinal cord transections made more caudally (closer to the mid-body or lower) can often swim immediately after transection. After 11 weeks recovery, we found that the 5th gill transected lampreys and the mid-body transected lampreys did not differ in any of the measured performance or kinematic parameters (Holm–Šidák *post hoc* comparison, *P*>0.05; Figs 1, 4 and 5). However, the mid-body transected lampreys had significantly lower *St* and stride lengths compared with controls (Holm–Šidák *post hoc* comparison, *P*>0.05; Fig. 6D).

## DISCUSSION

One of the primary goals of this study was to examine how swimming performance was related to degree of RS axon regeneration in lampreys recovering from spinal cord transection. We hypothesized that a larger fraction of RS axons regenerated would lead to more complete activation of the spinal locomotor circuits below the lesion owing to increased drive from the descending commands. Based on this hypothesis, we predicted that animals with a greater fraction of regenerated axons would swim faster and more efficiently than those with fewer regenerated axons. But that is not what we observed. Swimming speed was not related to the percent axon regeneration of lampreys recovered from spinal cord transection (Fig. 2). However, individuals swam at a range of speeds, whereby most individuals could swim both rapidly and slowly (Fig. S1). All the individuals had recovered for 10.5 weeks and had the ability to swim at a range of speeds and modulated their swimming speeds by changing their wave frequency and shape (Fig. 5). Despite being able to swim moderately fast at times, transected individuals did not produce body waves with as large amplitudes or as long wavelengths as the control lampreys. To swim rapidly, transected animals instead produced body waves at a high frequency, much higher than control animals used for swimming at the same speed. Transected animals also had higher Strouhal numbers and lower stride lengths. Together, these measurements suggest that the swimming efficiency was lower in the transected lampreys (both 5th gill and mid-body) compared with controls.

Our results suggest that there may be a limit to how much swimming performance lampreys can recover after spinal cord injury. All the transected lampreys in this study, regardless of percent axon regeneration, which ranged from 33 to 84%, had a similar relationship between their wave frequency and swimming speed (i.e. similar stride length; Fig. 6B). In fact, the wave kinematics and swimming performance of the 10.5 week recovered lampreys in this study were not much different than the 2 week recovered mid-body transected lampreys reported in Gemmell et al. (2016). It has been shown that spinal cord transected lampreys recover some locomotor function at 2 weeks, albeit with aberrant movements and locomotor activity, and appear to increase their locomotor activity after that (Davis et al., 1993; McClellan, 1994). By 8 weeks recovery, transected lampreys have near normal locomotor movement and muscle activity patterns (McClellan, 1994). However, others have shown that even after 10 weeks, recovered lampreys need to use higher wave frequencies than control lampreys to reach similar swimming speeds (Oliphint et al., 2010). Likewise, we found that the recovered, transected lampreys in this study also swam significantly shorter distances per tail beat than the control lampreys. This suggests that recovered, transected lampreys are not capable of coordinating the kinematics necessary to generate swimming thrust as efficiently as non-transected lampreys.

Why are control lampreys able to swim better than transected lampreys? Although all the lampreys in this study generated body waves that travel head to tail produced by waves of muscle activation on alternating sides of their body (McClellan et al., 2016; Williams et al., 1989), the shape and kinematics of these waves differed considerably between transected and control lampreys (Fig. 3). The body waves of the control lampreys had longer wavelength and higher amplitude, and the wave increased in amplitude as it traveled along the body (Fig. 4). In contrast, the wave amplitudes of the transected lampreys stayed fairly similar as the waves traveled along their bodies (Fig. 4). The gradual build-up of the wave amplitude has been shown to be essential for efficiently building and steering vortices for thrust generation (Gemmell et al., 2016). A comparison of the hydrodynamics generated by transected versus non-transected lampreys showed that the increase in wave amplitude gradually built up vorticity adjacent to the wave. The gradual build-up of vorticity led to the non-transected lampreys generating suction thrust consistently along most of the body (Gemmell et al., 2015, 2016). In contrast, the body waves of the transected lampreys did not increase in amplitude or build vorticity along the body, and thrust was inconsistent and primarily generated at the tail by positive pressure fields (Gemmell et al., 2016). This suggests that control lampreys get more thrust out of tail beat (Fig. 6).

We speculate that transected animals, although able to produce muscle activity, are not able to produce as forceful contractions as control animals. Lower muscle forces would result in lower amplitude body waves, as we observed (Fig. 2E). Similarly, computational work has suggested that when muscle forces are low compared with fluid forces, the body wavelength shortens (Tytell et al., 2010). If the wavelength of neural activity is similar in control and transected animals (as observed *in vitro* by McClellan, 1990), then the shorter mechanical body wavelength we observed would result in muscle activation earlier in the tail-beat cycle relative to muscle shortening, and thus more eccentric activity, particularly toward the tail. Such eccentric muscle activity does not produce propulsive power, but instead may stiffen the caudal region to more effectively transmit muscle force from the anterior body to the fluid (Blight, 1977; Tytell et al., 2010). However, if the anterior body is not producing force effectively, as seems to be occurring in transected animals, the body stiffening may not be useful and may instead reduce the total power produced, decreasing swimming efficiency. A limitation of the current study is that it does not enable us to understand the mechanisms that lead to decreased muscle forces in spinal-transected lampreys. This remains unclear as very little is known about the neural and muscular responses at the circuit level (Haspel et al., 2021). However, possibilities that may affect muscle forces include reduced input of descending commands to distal spinal circuits, including motor neurons and/or plasticity within the muscles themselves.

That lampreys can regain swimming behaviors post-recovery, despite incomplete axon regeneration, implies that other compensatory mechanisms are in play to restore locomotor behavior. In addition to RS axon regeneration, regeneration of other neuron types, as well as altered synaptic properties, has been observed within the lamprey spinal cord post-injury, which contribute to locomotor recovery (Becker and Parker, 2019; Cooke and Parker, 2009). Thus, the regenerated lamprey spinal cord is likely a ‘new’ locomotor network (Parker, 2017).

In conclusion, just as there appears to be more than one way to ‘skin a cat’, there appears to be more than one way for lampreys to swim. Recovered, transected lampreys clearly have the ability to swim and swim at high speeds. However, they have to produce many small body waves to achieve high swimming velocities, which control lampreys achieve using less frequent, larger waves. The differences in wave kinematics rely on different thrust mechanisms (Gemmell et al., 2016) and ultimately result in different swimming efficiencies.

## Supplementary Material

Supplementary information
