# Supplementary material for: Swimming kinematics and performance of spinal transected lampreys with different levels of axon regeneration
Source: J Exp Biol. 2021 Nov 5;224(21):jeb242639. doi: 10.1242/jeb.242639 (PMC8627570; doi:10.1242/jeb.242639)
Supplement: Supplementary information [file jexbio-224-242639-s1.pdf]

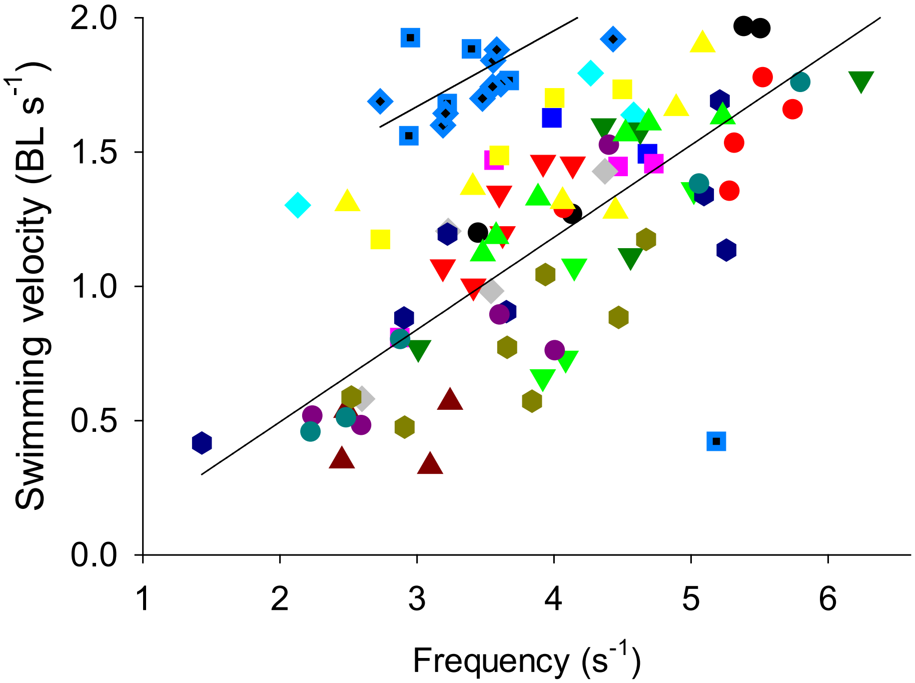

Fig. S1. Wave frequency versus swimming speed of individual swimming sequences. Replicate swim sequences of each individual are indicated by color. Blue are control lampreys with each individual having a different shape.

Table S1. Multiple regression for swimming speed as a function of tail beat frequency and regeneration percentage.

Effect	χ^2	*df*	*P*
**Frequency**	**77.9**	**1**	**<0.001**
Regeneration percentage	0.2	1	0.65
Frequency × Regeneration percentage	0.02	1	0.88

n = 83. Individual animal was included as a random effect in the model.
